# Multidrug resistance protein structure of *Trypanosoma evansi* isolated from buffaloes in Ngawi District, Indonesia: A bioinformatics analysis

**DOI:** 10.14202/vetworld.2021.33-39

**Published:** 2021-01-06

**Authors:** Moh. Mirza Nuryady, Raden Wisnu Nurcahyo, Iin Hindun, Diani Fatmawati

**Affiliations:** 1 Department of Biology Education, Faculty of Teacher Training and Education, Universitas Muhammadiyah Malang, Malang, Indonesia; 2 Master Program of Veterinary Science, Faculty of Veterinary Medicine, Universitas Gadjah Mada, Yogyakarta, Indonesia; 3 Department of Parasitology, Faculty of Veterinary Medicine, Universitas Gadjah Mada, Yogyakarta, Indonesia

**Keywords:** bioinformatics, buffaloes, multidrug resistance protein E, protein structure, Surra, *Trypanosoma evansi*

## Abstract

**Background and Aim:**

Trypanosomiasis, also known as surra, is an infectious disease with a wide host spectrum. In Indonesia, this disease is caused by *Trypanosoma evansi*. Various trypanocidal drugs have been used to treat this pathogen and subsequent disease. Yet, the long-term trypanocidal administration generates drug-resistant *T. evansi*. Some have identified genetic alterations in *T. evansi* transporter protein-coding genes that may be responsible for drug resistance. The Multidrug Resistance Protein E (MRPE) gene is a likely candidate gene responsible for the individual resistance. To date, no research has focused on *T. evansi* MRPE (*Tev*MRPE) in this context. Hence, this research aimed at analyzing and characterizing the *Tev*MRPE gene and protein using a bioinformatics approach.

**Materials and Methods:**

*T. evansi* was isolated from buffalo suffering from surra in Ngawi Regency, Indonesia. Isolated *T. evansi* was inoculated and cultured in male mice. The *T. evansi* genome was isolated from mouse blood with a parasitemia degree as high as 10^5^. A polymerase chain reaction procedure was conducted to amplify the putative MRPE coding gene. The amplicon was sequenced and analyzed using MEGA X, BLAST, and I-tasser softwares.

**Results:**

The putative *Tev*MRPE coding gene showed sequence similarity as high as 99.79% against the MRPE gene from *Trypanosoma brucei gambiense*. The protein profile and characteristics depicted that the putative *Tev*MRPE protein was related to a family of Adenosine Triphosphate-Binding Cassette (ABC) transporter proteins. This family of transporter proteins plays a crucial role in the resistance toward several medicines.

**Conclusion:**

The obtained gene sequence in this research was identified as the *Tev*MRPE. This gene is homologous to the *T*. *brucei gambiense* MRPE gene and possesses ligand active sites for Adenylyl Imidodiphosphate. In addition, MRPE contains enzyme active sites similar to the cystic fibrosis transmembrane conductance regulator. These data suggest that ABC transport proteins, like MRPE, may be necessary to confer trypanocidal drug resistance in *T. evansi*.

## Introduction

*Trypanosoma evansi* is a blood parasite that causes surra in a variety of hosts [1]. The reported parasite is not only deadly to cattle but also infects humans in some Asia countries [2]. The government typically uses trypanocidal drugs to combat surra. The trypanocides used today are similar to those first used in the 1920s: Suramin, isometamidium, diminazene, and quinapyramine dan melarsomine [3]. However, the long-term use of these drugs lead to a drug-resistant *T. evansi* infection [4,5]. Many studies in Indonesia have characterized resistant surra cases. Subekti *et al*. [6] reported that *T. evansi* isolated from different areas were resistant toward isometamidium. Later it was shown that *Trypanosoma* resistance was due to the decrease of transporter protein uptake function [7]. Consistent with that observation, a mutation in the *Trypanosoma brucei* Adenosine Transporter coding gene (*Tb*AT1) led to the *Trypanosoma* resistance toward diminazene aceturate [8]. Moreover, Delespaux *et al*. [9] reported the presence of a GAA codon insertion within the *Trypanosoma congolens* Adenosine Triphosphate (ATP)-Binding Cassette (ABC) Transporter coding gene to confer resistance to isometamidium. There are several other transporter protein families assumed to be responsible in *Trypanosoma* medicine resistance. One protein that is rarely considered is the multidrug resistance protein (MRP).

MRP is part of the ABC transporter family and is necessary for some resistance in *Trypanosoma* species toward trypanocides; particularly ivermectin, melarsoprol, and benznidazole [10,11]. The common MRP gene analyzed from *T. brucei* species is *Tb*MRPA [12]. To date, there is only one study focused on the MRP gene in *T. evansi* [13]. However, Nuryady *et al*. [13] only characterized and analyzed the phylogenetic profile of *T. evansi* MRP to other species. In addition, the primer sequences amplified regions of the MRPE gene that limits analyzing the family relationship among sub-species.

This study aimed to analyze the *T. evansi* MRPE (*Te*vMRPE) protein using *Tev*MRPE gene by bioinformatics. This bioinformatics analysis of the *Tev*MRPE coding gene is crucial to gain more detailed information about the characters and roles of this protein. This information will provide a deeper understanding of *Trypanosoma* medicine resistance.

## Materials and Methods

### Ethical clearance

All the experimental protocols employed in this research were examined and approved by the Veterinary Faculty Ethical Clearance Committee at Gadjah Mada University in Indonesia. Based on the approval letter No. 0024/EC-FKH/Int./2018, all procedures were conducted with strict adherence to the principles of laboratory animal care.

### Study period and location

This research was conducted from January 2018 to December 2020. The implementation of the research was carried out at the Parasitology Laboratory, Faculty of Veterinary Medicine, Universitas Gadjah Mada, Indonesia, Bioscience Laboratory of NODAI, Japan, and Biology Laboratory of Universitas Muhammadiyah Malang, Indonesia.

### Isolation and identification

The parasite was obtained from the pooled blood of 88 buffalo, which were positively identified with Surra in Ngawi Regency, Indonesia. The identification of *T. evansi* was conducted based on *in vivo* test and morphological structure. The *in vivo* test was applied by inoculating and culturing the parasite in 3-month-old male mice (*Mus musculus* strain Balb-C from Central University Laboratory UGM) by intraperitoneal injection. After 24 h, the mice were observed the parasitemia levels by drawing blood from the *vena coccygea* and observing the number also the morphology of *T. evansi* under a microscope (Olympus, CX22 series). Mice blood identified with more than 20 *T. evansi* in the microscope field view were recognized as a high level of parasitemia. In this condition, the mice were euthanatized, blood was taken, and stored at 4°C until DNA extraction.

### DNA extraction

DNA was extracted from mouse blood containing *T. evansi* using the DNA DNeasy Blood and Tissue kit (Qiagen, Hilden, Germany). The mouse blood was mixed with phosphate-buffered saline to a volume of 200μL. Proteinase K (20 μL) was added into the mixture and incubated at 60°C for 5 min using a shaker incubator. An equal volume of absolute alcohol was added to the mixture and the final mixture was loaded onto a GD column where the DNA was bound for subsequent washes (see company protocol for details). DNA was eluted from the GD column using 100 μL elution buffers.

### DNA amplification

A primer pair was developed from the MRPE *T. brucei* gene sequence: The reverse primer was 5’ATGAACGCTGACTCTGGTGA3’ and the forward primer was 5’GTAAGCAAG GCATTGTGGAA3’. A master mix was prepared with 25 μL 2× Go Taq Green polymerase Master Mix (Thermo Fisher Scientific, cot: K1081), 10 μL template DNA, primer (F:1 μL, R:1 μL), and distilled water to a total volume of 50 μL. Polymerase Chain Reaction (PCR) reactions were carried out on a Thermocycler (Labcycler Gradient, SensoQuest). The PCR reaction parameters were: Pre-denaturation at 94°C for 7 min, denaturation at 94°C for 30 s, annealing at 58°C for 30 s, extension at 72°C for 60 s, and termination at 72°C for 5 min for a total of 35 cycles.

### Sequencing and bioinformatics analysis

Sanger sequencing was conducted at First Base in Malaysia. DNA sequences were analyzed using MEGA 10 version and BLAST to obtain the protein composition and sub-species relationship. The protein composition was analyzed using I-tasser (https://zhanglab.ccmb.med.umich.edu/I-TASSER/) to predict the gene ontology, protein 3D structure, ligand binding sites, and active sites of the protein. The protein structure modeling obtained was used to compared homology to the other several related proteins. Structure differences were modeled using PyMol (Schrödinger, Inc., NY, USA).

## Results

### DNA sequence and phylogeny analysis

**Figure-1:**
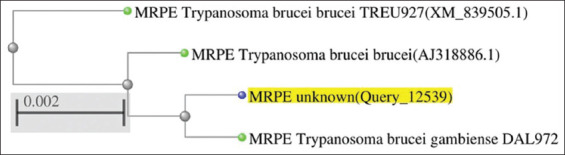
Phylogenetic diagram of multidrug resistance protein E *Trypanosoma evansi* analyzed using megablast.

Sanger sequencing was employed to characterize the putative *Tev*MRPE gene in relation to other sub-species. The gene was 939 nucleotides long. Blastx (MEGA X-highly similar sequence) was used to analyze the homologous relationship between sub-species. The alignment results showed that *Tev*MRPE sequence was identical to five sub-species (100% query coverage). The sequences were *Trypanosoma brucei gambiense* DAL972 MRPE (XM_011774747.1), *T. brucei gambiense* DAL972 chromosome 4 complete (FN554967.1), *Trypanosoma brucei brucei* mRNA MRPE (AJ318886.1), *T. brucei brucei* TREU927 MRPE (XM_839505.1), and *T. brucei* GUTat10.1 chromosome 4 complete (AC087327.7). In addition, the homologous identity compared to the MRPE *T. evansi* putative sequence and to MRPE *T. brucei gambiense* (XM_011774747.1) was 99.79%. The analysis results are summarized in Figure-1.

### Protein alignment of the putative TevMRPE gene

The putative *Tev*MRPE gene sequence was analyzed using the Blastx to get the amino acid sequence as well as the protein alignment against other sub-species. The analysis showed that there were five protein sequences with query coverage of at least 83%. These five proteins were classified into two groups. The first group consisted of *Trypanosoma brucei equiperdum* MRPA (RHW73256.1) with 100% homology. The other four MRPs (*T. brucei gambiense* MRPE [XP_011773049.1], *T. brucei brucei* MRPE [CAC83023.1], *Trypanosoma equiperdum* MRPA [SCU69648.1], and *T. brucei brucei* MRPE [XP_844598.1]) showed 99.6% homology. The results are shown in Table-1.

**Table-1:** Results from the protein alignment analysis of the *T. evansi* MRPE protein.

No.	Name of protein sequence	Accession No.	Query coverage
1.	MRPA of *Trypanosoma brucei equiperdum*	RHW73256.1	100%
2.	MRPE of *Trypanosoma brucei gambiense*	XP_011773049.1	99.6%
3.	MRPE of *Trypanosoma brucei brucei*	CAC83023.1	99.6%
4.	MRPA of *Trypanosoma equiperdum*	SCU69648.1	99.6%
5.	MRPE of *Trypanosoma brucei brucei*	XP_844598.1	99.6%

*T. evansi* MRPE= *Trypanosoma evansi* Multidrug Resistance Protein E

### Conserved protein domain analysis

**Figure-2:**
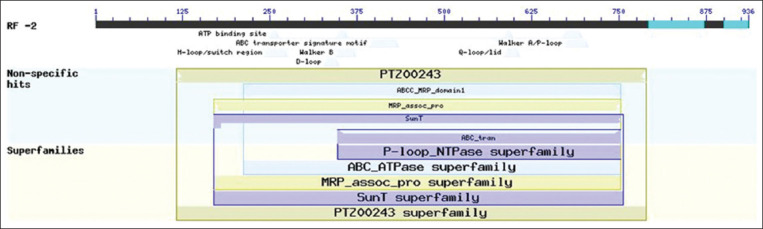
The results analysis of conserved protein using online Blast.

As shown in Figure-2, the protein sequence translated from the putative *Tev*MRPE gene aligns with many proteins from the ABC transporter superfamily (PTZ00243): *A*BC domain 1 multidrug resistance-associated protein subfamily C (cd03250), multidrug resistance-associated protein MRP (TIGR00957), ABC-type bacteriocin/antibiotic exporters (COG2274), and ABC Transporter (pfam00005).

### Predicting the putative TevMRPE protein using bioinformatics analysis

To predict the *Tev*MRPE protein template, protein threading was performed by aligning the *Tev*MRPE protein target sequence with similar template protein sequences obtained from the Protein Data Bank (PDB, i.e., structure of the human MRP 1 Nucleotide Binding Domain 1 [NBD1]); 6BZS (Human ABCC6 NBD1 in Apo state); 6BZS (Human ABCC6 NBD1 in Apo state); 3WME (Crystal structure of an inward-facing eukaryotic ABC multidrug transporter); 1R0Z (Phosphorylated Cystic fibrosis transmembrane conductance regulator (CFTR) NBD1 with ATP; 5U71 (Structure of human CFTR); 6BZS [Human ABCC6 NBD1 in Apo state]; and 1Q3H [mouse CFTR NBD1 with AMP.PNP]). PDB templates that align well with the target protein are assumed to possess the similar protein structures [14]. The protein threading results showed that there were three template sequences that resembled the *Tev*MRPE protein structure (Table-2). These three templates were 2cbz (Structure of the human MRP 1 NBD1); 6bzs (Human ABCC6 NBD1 in Apo state); and 6bzs (Human ABCC6 NBD1 in Apo state).

**Table-2:** The protein threading results of the *T. evansi* MRPE protein.

No.	PDB hit	Coverage	Norm. Z-score	Protein name	Function
1.	2cbzA	0.84	3.36	Human MRP 1 nucleotide-binding domain 1	Transport protein
2.	6bzsA	0.83	3.85	Human ABCC6 nucleotide-binding domain 1 in Apo state	Transport protein
3.	6bzsA	0.83	4.06	Human ABCC6 nucleotide-binding domain 1 in Apo state	Transport protein

- Coverage represents the coverage of the threading alignment and is equal to the number of aligned residues divided by the length of query protein. Norm. Z-score is the normalized Z-score of the threading alignments. Alignment with a Normalized Z-score >1 mean a good alignment and *vice versa. T. evansi* MRPE=*Trypanosoma evansi* Multidrug Resistance Protein E

### Prediction model of the TevMRPE protein structure

**Figure-3:**
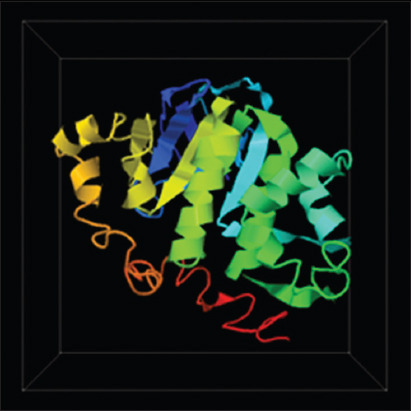
The prediction model of the highest C-score (−0.66) *Trypanosoma evansi* Multidrug resistance protein E structure

Five protein models were generated based on the modeling prediction results. However, the first model was the closest representation of MRPE, possessing the highest C-score (−0.66), estimated TM-score (0.63±0.14), and root-mean-square deviation (RMSD) (7.3±4.2 Å) (Figure-3).

**Figure-4:**
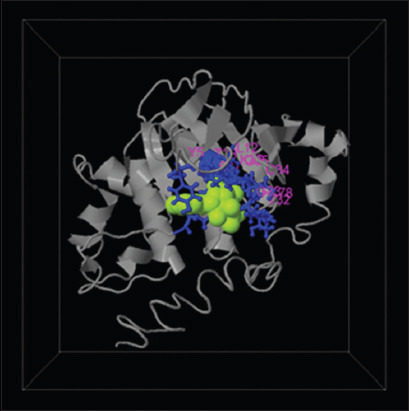
The prediction structure of the highest C-score ligand site (0.8) in *Trypanosoma evansi* multidrug resistance protein E.

Five predicted protein ligand sites were identified. The ligand site with the highest C-score (0.8) was Adenylyl Imidodiphosphate (ANP-PMP). The modeling prediction results of the five ligand sites are shown in Table-3. The ligand site prediction of the *Tev*MRPE protein is found in Figure-4.

**Table-3:** The ligand name prediction and residual binding sites of the *T. evansi* MRPE protein.

Rank	C-score	Ligand name	Ligand binding site residues
1	0.81	ANP PMP	5, 12, 32, 33, 34, 35, 36, 37, 38, 66, 178
2	0.17	NA	37, 66, 145, 146
3	0.15	ATP	120, 121, 122, 123, 124, 125
4	0.02	128	5, 10, 12, 33, 34, 35, 36, 37, 38, 41, 48
5	0.01	ALF	32, 33, 36, 66, 146, 178

C-score is the confidence score of the prediction. C-score ranges (0-1), where a higher score indicates a more reliable prediction. *T. evansi* MRPE=*Trypanosoma evansi* Multidrug Resistance Protein E, ALF=Aluminate ion, ATP=Adenosine triphosphate, ANP PMP=Adenylyl imidodiphosphate

### Enzyme commission (EC) prediction and its active site

**Figure-5:**
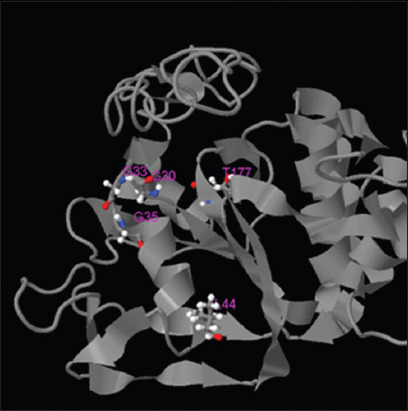
The prediction structure of enzyme commission active site in *Trypanosoma evansi* multidrug resistance protein E.

An EC number is a numeric classification based on the chemical reaction catalyzed. The closest model enzyme (Model 1; C-score, 0.414; and TM-score, 0.804) relating to the *Tev*MRPE protein was classified as a transport protein (EC number 3.6.3.49), which has five active sites for enzyme binding (Figure-5).

### Gene ontology prediction

Gene ontology predicts and/or identifies the molecular function, biological process, and cellular location of the *Tev*MRPE protein (Table-4). The two highest molecular function predictions are ATP Binding and ATPase-coupled transmembrane transporter activity with GO-scores of 0.95 and 0.48, respectively. This protein is involved in biological processes such as transmembrane transport and obsolete ATP catabolic process with GO-scores of 0.48, each. This protein is suggested to be located in the plasma membrane and membrane integral component with GO-scores of 0.48 for both.

**Table-4:** The gene ontology of the *T. evansi* MRPE protein.

Molecular function	GO-score	Biological process	GO-score	Cellular component	GO-score
ATP-binding (GO:0005524)	0.95	Transmembrane transport (GO:0055085)	0.48	Plasma membrane (GO:0005886)	0.48
ATPase-coupled transmembrane transporter activity (GO:0042626)	0.48	Obsolete ATP catabolic process (GO:0006200)	0.48	Integral component of membrane (GO:0016021)	0.48

The GO-score is a combined measure for evaluating global and local similarity between query and template protein. Its range is 0-1 and higher values indicate more confident predictions. *T. evansi* MRPE=*Trypanosoma evansi* Multidrug Resistance Protein E, ATP=Adenosine triphosphate

## Discussion

The primers were designed from *T. brucei* because there are no reports about MRPE gene of *T. evansi* in the GenBank and due to the research result [1] that *T. brucei* has a closer relationship with *T. evansi* than other species. The result of the primer blast analysis showed that the primer was specific to the *T. brucei* MRPE gene only. The primer was expected to amplify an amplicon containing 1116 nucleotides. However, the sequence BLAST analysis demonstrated that the MRPE gene is 936 nucleotides long, suggesting the actual gene is shorter than the predicted length. The most similar gene determined by NCBI blast was the *T. brucei gambiense* MRPE gene, DAL972 (XM_011774747.1). Its sequence similarity percentage was as high as 99.79% (Figure-1). This finding is in agreement with the previous research that reported a relationship between *T. brucei gambiense* and *T. evansi* based on their MRPE gene sequences [13]. This observation is consistent with the idea that *T. brucei* has evolved, bearing several sub-species such as *T. brucei brucei*, *T. brucei equiperdum, T. brucei gambiense*, and *Trypanosoma brucei evansi* [15]. Furthermore, the evolution of these sub-species was found in the mitochondrial genes of *T. brucei* spp. [1,16].

Likewise, the protein alignment results of the *Tev*MRPE protein depicted a high similarity percentage (100%) with the *Trypanosoma brucei equiperdum* MRPA protein (Table-1). *T. evansi* belongs to the same clade of *T. brucei* sub-species. Interestingly, *T. evansi* and *T. brucei* genomes are not that similar as the only overlapping sequences between the two are contained to one minicircle of genetic material [17,18]. In contrast, the differences between *T. equiperdum* and *T. evansi* are contained in maxicircle kDNA. The maxicircle found in *T. equiperdum* is bigger compared to *T. evansi*’s, suggesting it was reduced by a deletion process [19].

The *Tev*MRPE putative gene had high similarity to the *T. equiperdum* MRPA gene, suggesting that these genes are well conserved at the sub-species level [20]. Many studies have identified that MRPE is a C type ABC transporter characterized by a long N-terminus hydrophilic transmembrane domain, which possesses a protein region of high homology [21-24]. The putative *Tev*MRPE protein sequence in Figure-2 showed that there are five conservative areas among the ABC transporter family; the ABC transporter signature motif, the H-Loop/switch region, the Walker B D-Loop, the Walker A/P-loop, and the Q-loop [25,26]. These domains are important for identifying ABC transporters among other transporter proteins.

The threading protein prediction results (Table-2) showed that the *Tev*MRPE protein had high similarity to three template sequences. The three proteins were the Human MRP 1 NBD1 (2CBZ) protein, the Human ABCC NBD1 in Apo-state (6BZS) protein, and the *Cyanidioschyzon merolae* ABC multidrug transporter protein. All of these template proteins are ABC transporter proteins. This was no surprise as the ABC transporter protein is the largest protein superfamily that is well conserved across all organism [27]. Of the five protein models compared to the MRPE structure, the first model was (2CBZ) the best representation with an estimated TM-score of 0.63±0.14 and estimated RMSD of 7.3±4.2 Å. As no previous MRPE *T. evansi* protein model published in PDB, the five protein models were the closest model resemble the MRPE of *T. evansi*.

The ligand site prediction showed five ligand sites: ANP, Sodium ion (NA), ATP, 128 Spiro (2,4,6-trinitrobenzene [1,2a]-2o’,3o’- methylene- adenine- triphosphate), and Tetrafluoro aluminate ion. The five predicted ligand sites are commonly found in transporters (Table-3 and Figure-4). These findings agree with the previous reporting that MRPs play a crucial role as active transporters with three potential membrane-spanning domains [28,29]. Conserved gene organization and protein structure features suggest that MRP and its related proteins share a similar ancestry with the cystic fibrosis conductance regulator [30,31].

The ANP ligand site has an analog function with ATP-binding oxygen atoms with beta- and gamma-phosphate groups. This enables ANP to serve as a potential competitive mitochondrial ATPase inhibitor, the major protein that functions in ATP production and regulated by oxidative phosphorylation output [32].

The predicted EC number results showed that the putative MRPE protein is strongly related to other transmembrane transporters involved in trypanocidal resistance. The most similar transmembrane transport protein identified was 1r0zC, which is known as the phosphorylated CFTR containing NBD1. CFTR is an ABC transporter that functions as a chloride channel [33,34]. NBD1, one of two ABC domains in CFTR, also contains sites for cystic fibrosis causing mutations in regulatory phosphorylation sites [35].

Gene ontology prediction showed that MRPE’s main function is as a transmembrane transporter and is located in cell plasma membranes (Table-4). This information is consistent with the previous ABC transporter protein structure reports [36]. These proteins are composed of two transmembrane hydrophobic (TMD) domains involved in substrate translocation. In addition, the NBD is responsible for ATP binding and hydrolysis.

The main function of an ABC transporter protein is exporting various cellular materials outside of the cell. Baker *et al*. [7] show that as melarsoprol penetrated *Trypanosoma* cells, the increase of ABC transporter work increased. Likewise, Delespaux *et al*. [9] found that isometamidium resistance in *T. congolens* is determined by the insertion of GAA codon in ABC transporter coding gene. These data and our study support the idea that ABC transport proteins in *T. evansi* are necessary for the bacteria’s resistance to many drugs. Some of the MRP members have five additional transmembrane segments in their N-terminus, but the function of these additional membrane-spanning domains is not clear. Typically, MRP exports glutathione, glucuronate, and sulfate from specific drug stimulation [31,37]. Thus, MRP may be a transporter protein that plays an active role in increasing the number of trypanocidal drug resistance cases, especially with cases using thiol conjugates, ivermectin, melarsoprol and benznidazole [10,11].

## Conclusion

This study isolated the gene MRPE, also known as T*ev*MRPE, from *T. evansi* is homologous to the *T. brucei gambiense* MRPE gene. This gene is predicted to have an active ligand site for AMP-PMP and active enzyme site similar to that of the CFTR. These ABC transporter superfamily characteristics may be important for the transporting functions responsible for trypanocidal drug resistance in *T. evansi*.

## Authors’ Contributions

RWN planned the entire research work. MMN and DF carried out the laboratory work and sample collection and analyzed the Bioinformatic data. IH administered the research work and analyzed the Bioinformatic data. All authors have read and approved the final manuscript.

## References

[1] Desquesnes M, Holzmuller P, Lai D.H, Dargantes A, Lun Z.R, Jittaplapong S (2013). *Trypanosoma evansi* and surra: A review and perspectives on origin, history, distribution, taxonomy, morphology, hosts, and pathogenic effects. Biomed. Res. Int.

[2] van Vinh Chau N.V.V, Chau L.B, Desquesnes M, Herder S, Lan N.P.H, Campbell J.I, van Cuong N, Yimming B, Chalermwong P, Jittapalapong S, Franco J.R, Tue N.T, Rabaa M.A, Carrique-Mas J, Thanh T.P.T, Vu Thieu N.T, Berto A, Hoa N.T, van Minh Hoang N, Tu N.C, Chuyen N.K, Wills B, Hien T.T, Thwaites G.E, Yacoub S, Baker S (2016). A clinical and epidemiological investigation of the first reported human infection with the zoonotic parasite *Trypanosoma evansi* in Southeast Asia. Clin. Infect. Dis.

[3] Subekti D.T (2014). Perkembangan, struktur, mekanisme kerja dan efikasi trypanosidal untuk surra (Development, structure, mechanism of action and efficacy of trypanocidal for surra). Wartazo a.

[4] Geerts S, Holmes P.H, Eisler M.C, Diall O (2001). African bovine trypanosomiasis: The problem of drug resistance. Trends Parasitol.

[5] Ponnudurai G, Sivaraman S, Rani N, Veerapandian C (2015). An outbreak of trypanosomosis in buffaloes caused by diminazene resistant *Trypanosoma evansi*. Buffalo Bull.

[6] Subekti D.T, Yuniarto I, Sulinawati S, Susiani H, Amaliah F, Santosa B (2015). Trypanocidals effectivity against some isolates of *Trypanosoma evansi* propagated in mice. J. Ilmu Ternak Vet.

[7] Baker N, de Koning H.P, Mäser P, Horn D (2013). Drug resistance in African trypanosomiasis:The melarsoprol and pentamidine story. Trends Parasitol.

[8] Medina N.P, Mingala C.N (2016). Transporter protein and drug resistance of *Trypanosoma*. Ann. Parasitol.

[9] Delespaux V, Geysen D, Majiwa P.A.O, Geerts S (2005). Identification of a genetic marker for isometamidium chloride resistance in *Trypanosoma congolense*. Int. J. Parasitol.

[10] Ardelli B.F (2013). Transport proteins of the ABC systems superfamily and their role in drug action and resistance in nematodes. Parasitol. Int.

[11] Campos M.C, Phelan J, Francisco A.F, Taylor M.C, Lewis M.D, Pain A, Clark T.G, Kelly J.M (2017). Genome-wide mutagenesis and multidrug resistance in American trypanosomes induced by the front-line drug benzimidazole. Sci. Rep.

[12] Alibu V.P, Daunes S, D'Silva C (2013). N-benzyloxycarbonyl-S-(2, 4-dinitrophenyl) glutathione dibutyl diester is inhibitory to melarsoprol resistant cell lines overexpressing the *T. brucei* MRPA transporter. Bioorg. Med. Chem. Lett.

[13] Nuryady M.M, Widayanti R, Nurcahyo R.W, Fadjrinatha B, Fahrurrozi Z.S.A (2019). Characterization and phylogenetic analysis of multidrug-resistant protein-encoding genes in *Trypanosoma evansi* isolated from buffaloes in Ngawi district, Indonesia. Vet. World.

[14] Xu J, Jiao F, Yu L (2008). Protein structure prediction using threading. Methods Mol. Biol.

[15] Kamidi C.M, Saarman N.P, Dion K, Mireji P.O, Ouma C, Murilla G, Aksoy S, Schnaufer A, Caccone A (2017). Multiple evolutionary origins of *Trypanosoma evansi* in Kenya. PLoS Negl. Trop. Dis.

[16] Hong X.K, Zhang X, Fusco O.A, Lan Y.G, Lun Z.R, Lai D.H (2017). PCR-based identification of *Trypanosoma lewisi* and *Trypanosoma musculi* using maxicircle kinetoplast DNA. Acta Trop.

[17] Molinari J, Moreno S.A (2018). *Trypanosoma brucei* Plimmer and Bradford, 1899 is a synonym of *T evansi* (Steel 1885) according to current knowledge and by application of nomenclature rules. Syst. Parasitol.

[18] Cooper S, Wadsworth E.S, Ochsenreiter T, Ivens A, Savill N.J, Schnaufer A (2019). Assembly and annotation of the mitochondrial minicircle genome of a differentiation-competent strain of *Trypanosoma brucei*. Nucleic Acids Res.

[19] Lun Z.R, Li A.X, Chen X.G, Lu L.X, Zhu X.Q (2004). Molecular profiles of *Trypanosoma brucei*, *T evansi* and *T. equiperdum* stocks revealed by the random amplified polymorphic DNA method. Parasitol. Res.

[20] Coelho A.C, Cotrim P.C (2018). The role of ABC transporters in drug-resistant *Leishmania.*. Drug Resistance in Leishmania Parasites.

[21] Sauvage V, Aubert D, Escotte-Binet S, Villena I (2009). The role of ATP-binding cassette (ABC) proteins in protozoan parasites. Mol. Biochem. Parasitol.

[22] Chen Z, Shi T, Zhang L, Zhu P, Deng M, Huang C, Hu T, Jiang L, Li J (2016). Mammalian drug efflux transporters of the ATP binding cassette (ABC) family in multidrug resistance:A review of the past decade. Cancer Lett.

[23] El-Awady R, Saleh E, Hashim A, Soliman N, Dallah A, Elrasheed A, Elakraa G (2016). The role of eukaryotic and prokaryotic ABC transporter family in failure of chemotherapy. Front. Pharmacol.

[24] Fairlamb A.H, Horn D (2018). Melarsoprol resistance in African trypanosomiasis. Trends Parasitol.

[25] Eckenstaler R, Benndorf R.A (2020). 3D structure of the transporter ABCG2-what's new?. Br. J. Pharmacol.

[26] Remali J, Aizat W.M, Ng C.L, Lim Y.C, Mohamed-Hussein Z.A, Fazry S (2020). *In silico* analysis on the functional and structural impact of Rad50 mutations involved in DNA strand break repair. PeerJ.

[27] Zhang Y (2008). I-TASSER server for protein 3D structure prediction. BMC Bioinformatics.

[28] Costa K, Salustiano E.J, Valente R, Lima L.F.D, Mendonca-Previato L, Previato J.O (2020). Thiol Efflux Mediated by an ABCC-Like Transporter Participates for *Trypanosoma cruzi* Adaptation to Environmental and Chemotherapeutic Stresses, bioRxiv.

[29] Téllez J, Romero I, Romanha A.J, Steindel M (2019). Drug transporter and oxidative stress gene expression in human macrophages infected with Benznidazole-sensitive and naturally Benznidazole-resistant *Trypanosoma cruzi* parasites treated with Benznidazole, Parasit. Vectors.

[30] Deeley R.G, Cole S.P.C (1997). Function, evolution and structure of multidrug resistance protein (MRP). Semin. Cancer Biol.

[31] Leslie E.M, Deeley R.G, Cole S.P.C (2005). Multidrug resistance proteins:Role of P-glycoprotein, MRP1, MRP2, and BCRP (ABCG2) in tissue defense. Toxicol. Appl. Pharmacol.

[32] Shafqat N, Muniz J.R, Pilka E.S, Papagrigoriou E, von Delft F, Oppermann U, Yue W.W (2013). Insight into S-adenosylmethionine biosynthesis from the crystal structures of the human methionine adenosyltransferase catalytic and regulatory subunits. Biochem. J.

[33] Liu F, Zhang Z, Csanády L, Gadsby D.C, Chen J (2017). Molecular structure of the human CFTR ion channel. Cell.

[34] Linsdell P (2018). Cystic fibrosis transmembrane conductance regulator (CFTR):Making an ion channel out of an active transporter structure. Channels (Austin).

[35] Lewis H.A, Buchanan S.G, Burley S.K, Conners K, Dickey M, Dorwart M, Fowler R, Gao X, Guggino W.B, Hendrickson W.A, Hunt J.F, Kearins M.C, Lorimer D, Maloney P.C, Post K.W, Rajashankar K.R, Rutter M.E, Sauder J.M, Shriver S, Thibodeau P.H, Thomas P.J, Zhang M, Zhao X, Emtage S (2004). Structure of nucleotide-binding domain 1 of the cystic fibrosis transmembrane conductance regulator. EMBO J.

[36] Schneider E, Hunke S (1998). ATP-binding-cassette (ABC) transport systems:Functional and structural aspects of the ATP-hydrolyzing subunits/domains. FEMS Microbiol. Rev.

[37] Johnson Z.L, Chen J (2017). Structural basis of substrate recognition by the multidrug resistance Protein MRP1. Cell.

